# Intramural Esophageal Hematoma Secondary to Food Ingestion

**DOI:** 10.7759/cureus.5623

**Published:** 2019-09-11

**Authors:** Bashar Sharma, Dhruv Lowe, Marsha Antoine, Mili Shah, Ronald Szyjkowski

**Affiliations:** 1 Internal Medicine, State University of New York Upstate Medical University, Syracuse, USA; 2 Gastroenterology, State University of New York Upstate Medical University, Syracuse, USA

**Keywords:** esophageal wall injury, food ingestion, esophageal hematoma

## Abstract

Intramural esophageal hematoma (IEH) is a rare cause of submucosal esophageal bleeding and it is on the spectrum of esophageal wall injury along with mucosal tears (Mallory-Weiss syndrome) and full thickness perforation (Boerhaave’s syndrome). Its risk factors include coagulopathy, trauma (foreign body ingestion or esophageal instrumentation) or it can happen spontaneously. It presents with a triad of chest pain, dysphagia, and hematemesis; however, the triad is only present in 35% of patients. We are presenting a case of IEH secondary to food ingestion that was managed successfully by conservative measures.

## Introduction

Intramural esophageal hematoma (IEH) is a rare cause of submucosal esophageal bleeding. It is on the spectrum of esophageal wall injury along with mucosal tears (Mallory-Weiss syndrome) and full thickness perforation (Boerhaave’s syndrome) [1]. It can be precipitated by coagulopathy, trauma (foreign body ingestion or food impaction), forceful vomiting or retching [2]. It can also be iatrogenic usually secondary to esophageal instrumentation [3] or it can happen spontaneously [4]. It presents with a triad of retrosternal chest pain, dysphagia, and hematemesis; however, the triad is only seen in 35% of patients [2]. Diagnosis can be missed as the presentation can be confused with cardiopulmonary diseases; thus, a high index of suspicion is crucial. Here we are presenting a case of IEH secondary to food ingestion.

## Case presentation

**Figure 1:**
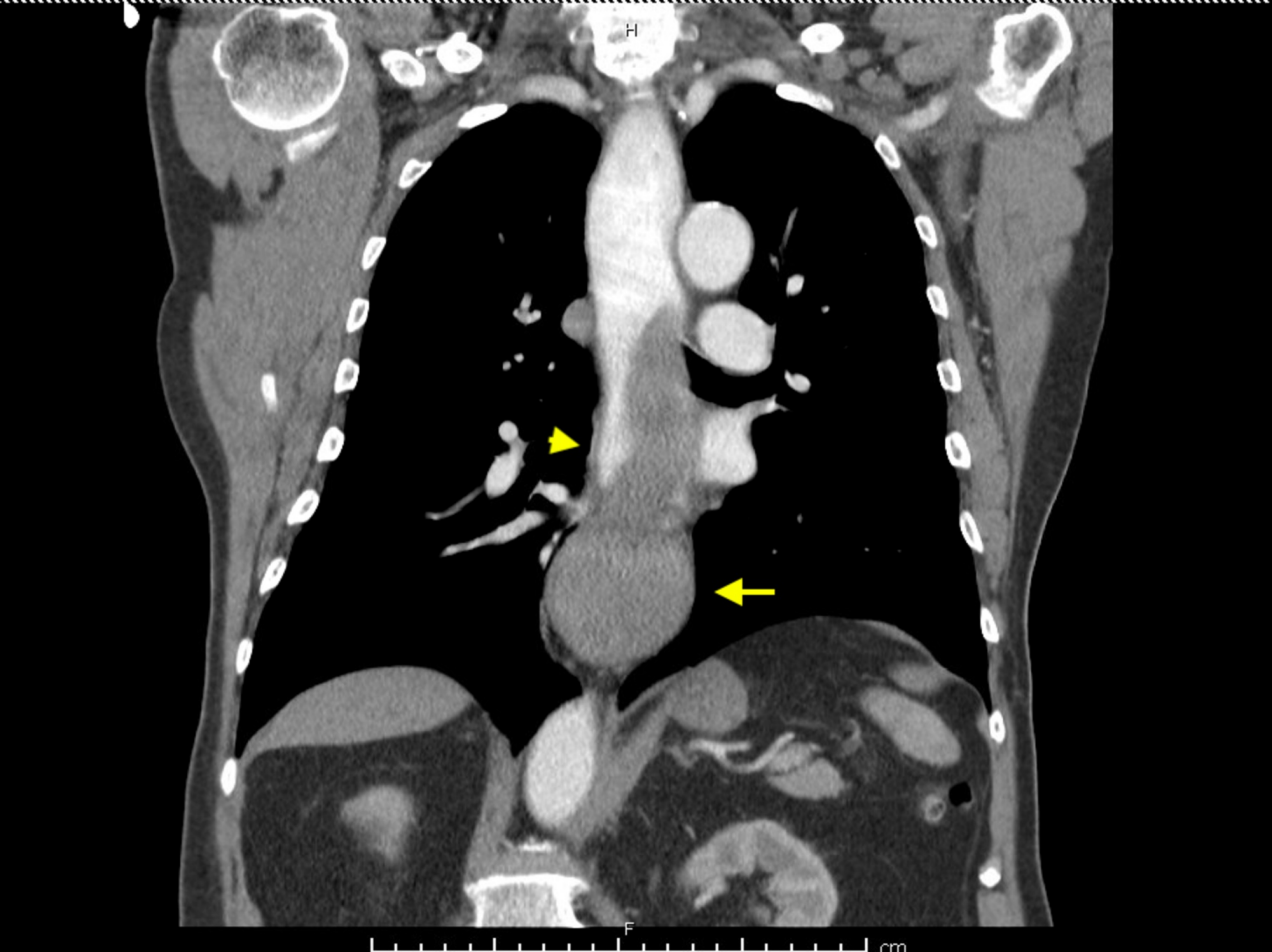
Computed tomography (CT) thorax with oral and intravenous (IV) contrast showing esophageal luminal narrowing at the distal third (yellow arrowhead) with mural thickening and a soft tissue density extending to the gastroesophageal junction (yellow arrow)

**Figure 2:**
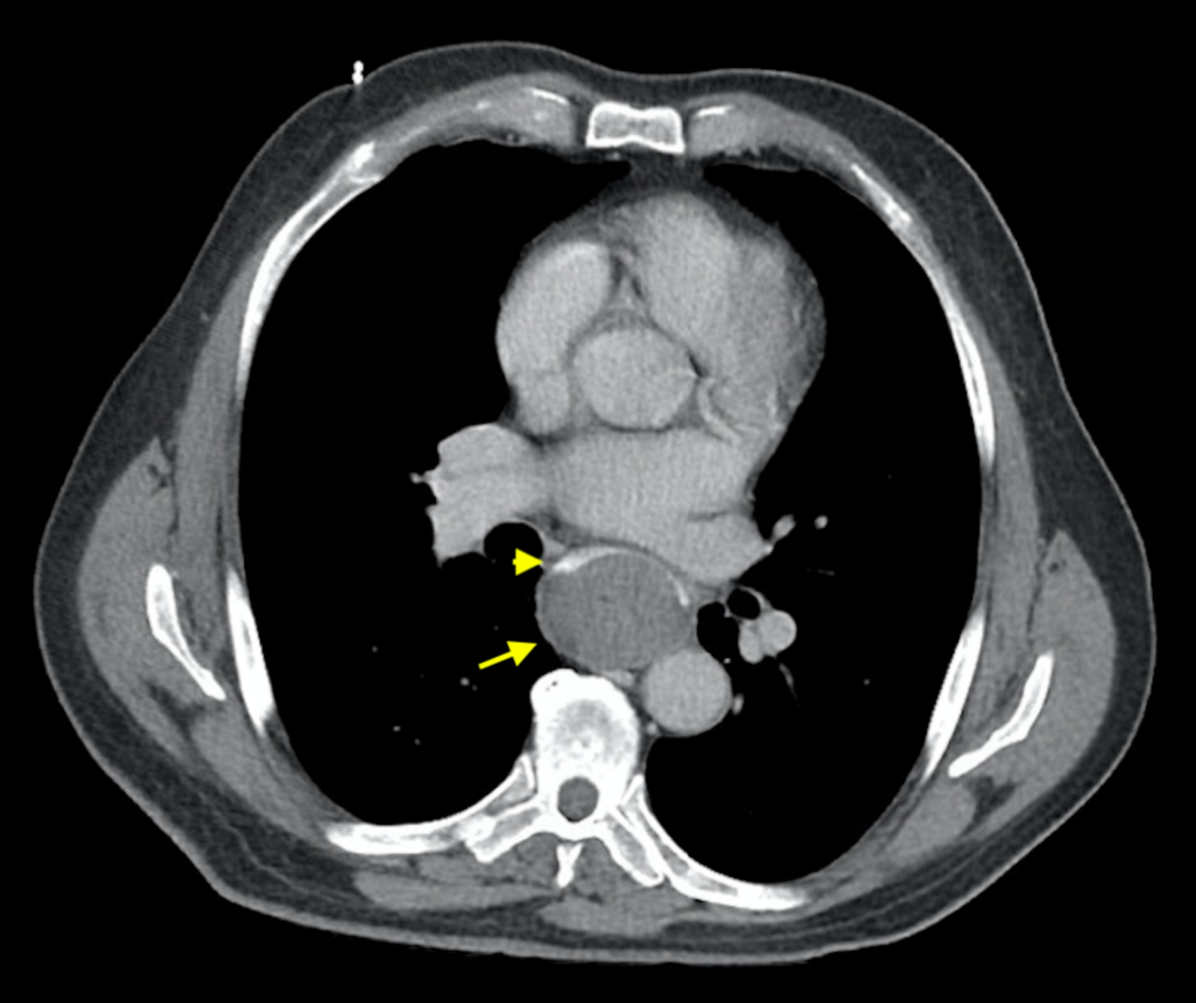
Computed tomography (CT) abdomen with oral and intravenous (IV) contrast showing hyperdense oral contrast layering (yellow arrowhead) over a rounded, well-circumscribed structure in the visualized distal esophagus (yellow arrow) concerning for an intramural esophageal hematoma

A 75-year-old male presented with worsening of his dysphagia, retrosternal chest pain and hematemesis. One day prior to admission, he was eating a fish sandwich and developed acute onset dysphagia with difficulty swallowing food and his own saliva after a few bites. Next morning, he developed sudden onset sharp retrosternal chest pain associated with hematemesis of bright red blood so he presented to the hospital. He denied having any odynophagia, abdominal pain, nausea or fever. On admission, his blood pressure was 130/82 mmHg and pulse rate 90 bpm. Physical examination was unremarkable. Laboratory workup showed hemoglobin 12.5 g/dl, hematocrit 40.3%, platelets 308,000/uL with normal coagulation profile. His blood urea nitrogen (BUN) was 25 mg/dL with serum creatinine 0.7 mg/dL. Electrocardiogram (EKG) was unremarkable. Computed tomography (CT) thorax with oral and intravenous (IV) contrast showed esophageal luminal narrowing at the distal third with mural thickening and a soft tissue density extending to the gastroesophageal junction (GEJ) concerning for esophageal hematoma (Figure 1). CT abdomen with oral and IV contrast showed hyperdense oral contrast layering over a rounded, well-circumscribed hyperdense structure in the visualized distal esophagus concerning for an intramural esophageal hematoma extending inferiorly to the level of the GEJ (Figure 2).

**Figure 3:**
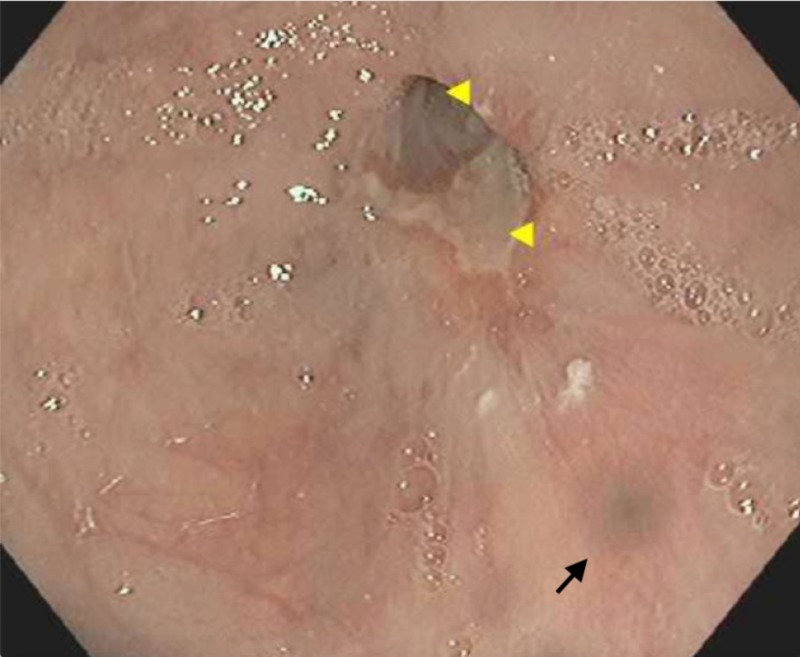
Esophagogastroduodenoscopy showing bluish discoloration at the distal third of the esophagus (black arrow) and a non-bleeding ulcer at the gastroesophageal junction (yellow arrowheads)

He was kept nothing by mouth (NPO) at first and started on IV fluids and pantoprazole infusion. Subsequently, his symptoms improved with conservative management and his diet was advanced to clear liquid diet. Four days after admission, he underwent an esophagogastroduodenoscopy (EGD) which showed a bluish discoloration at the distal third of the esophagus and a non-bleeding ulcer at the GEJ (Figure 3). No biopsies were taken given his recent bleeding and hematoma. He was started on a pureed diet with oral pantoprazole 40 mg twice daily, which he tolerated well and was discharged home. He did not have any recurrence of his symptoms.

## Discussion

IEH is an uncommon condition characterized by submucosal bleeding. It is most commonly seen in the distal third of the esophagus as it is less supported due to the absence of striated muscle and adjacent structures such as the trachea and the heart [5]. It is more common in females and its incidence tends to increase with age [5-6]. It can be triggered by a sudden change in intrathoracic and intraesophageal pressure such as during forceful vomiting, retching or coughing [7]. Use of anticoagulants, antiplatelets, and coagulopathic disorders has also been associated with the development of IEH [6,8-9]. Direct trauma to the esophagus caused by instrumentation or foreign body ingestion can precipitate it as well [3,10]. IEH has also been reported spontaneously without any clear etiology or any of the above risk factors [4]. In our case, food ingestion likely resulted in direct trauma to the esophagus.

The classic presentation is a triad of sudden onset retrosternal chest pain, dysphagia/odynophagia, and hematemesis; however, the triad is only seen in 35% of patients with about 80% of patients presenting with two of the three symptoms [11]. Physical examination is non-specific, yet, it is important to look for crepitus at the neck and upper chest that can be seen with perforation. Evaluation involves ruling out other differential diagnoses that present with acute chest pain, especially cardiopulmonary diseases. Complete blood count and coagulation profile should be checked and anticoagulation/antiplatelets should be held.

IEH can be diagnosed with a number of imaging modalities including Barium swallow, CT, magnetic resonance imaging (MRI), EGD or endoscopic ultrasound (EUS). Barium swallow can show a longitudinal intraluminal filling defect along with the normal esophageal lumen 'double-barreled esophagus' or ‘mucosal strip sign’ [12-13]. CT should be considered as the initial investigation of choice as it is noninvasive and can rule out other thoracic diseases or involvement of surrounding structures. It can show symmetric or asymmetric esophageal wall thickening with intramural soft tissue density that extends along the esophageal wall [13] as seen in our case. Measurement of attenuation of the hematoma will correspond to blood density that varies based on the age of the hematoma [13]. Addition of oral contrast helps in visualizing the narrowed esophageal lumen as well as in ruling out perforation. An early upper endoscopy can be considered especially if hematemesis is the primary concern [14]; however, esophageal integrity should be confirmed first. Typical findings include obliteration of the esophageal lumen and visualization of a bluish longitudinal mass with a friable mucosa with or without a visible tear [13-14]. EUS may help in confirming the diagnosis by showing a homogeneously hypoechoic lesion in the submucosa [13].

Most cases usually resolve with conservative measures with a resolution of symptoms in 1-2 weeks. Conservative measures include NPO, IV fluids, acid suppression, and correction of coagulopathy if present [8]. A soft diet may be started in stable patients and progressively advanced based on symptom improvement. Surgical intervention is rarely needed and usually reserved for patients with massive hemorrhage and/or become hemodynamically unstable [15].

In our case, the diagnosis was confirmed with CT. EGD showed a localized area of mucosal skin discoloration which we believe was the site of the direct trauma by the ingested food (as seen in Figure 3). The luminal narrowing was not as severe as seen in the CT likely due to early resolution of the hematoma and explains the improvement of the symptoms. He was managed successfully with conservative management and was discharged on a pureed diet within one week of the presentation without recurrence of his symptoms.

## Conclusions

In conclusion, IEH is a rare cause of submucosal esophageal injury that can present with acute onset chest pain, dysphagia or hematemesis, mimicking other conditions especially cardiopulmonary diseases. This can result in a delayed or even missed diagnosis. Thus, a high index of suspicion is needed to make the diagnosis and provide the appropriate management.
